# Engaging people with lived experience in the grant review process

**DOI:** 10.1186/s12910-019-0436-0

**Published:** 2019-12-16

**Authors:** Katherine Rittenbach, Candice G. Horne, Terence O’Riordan, Allison Bichel, Nicholas Mitchell, Adriana M. Fernandez Parra, Frank P. MacMaster

**Affiliations:** 1 Addiction and Mental Health Strategic Clinical Network, Alberta Health Services, Alberta Children’s Hospital, 2888 Shaganappi Trail NW, Calgary, AB T3B 6A8 Canada; 2 1E6.13 Department of Psychiatry, WMC Health Sciences Centre, Edmonton, AB T6G 2B7 Canada; 3 Centre Suite 110 Stn 1423N 200 4th Avenue, South Lethbridge AB, T1J4C9 Canada; 4 10101 Southport Road SW, Calgary AB, T2E 6Z8 Canada; 5 Alberta Children’s Hospital, 2888 Shaganappi Trail NW, Calgary, AB T3B 6A8 Canada

**Keywords:** Funding, Grant, Lived experience, Patient engagement, Review

## Abstract

People with lived experience are individuals who have first-hand experience of the medical condition(s) being considered. The value of including the viewpoints of people with lived experience in health policy, health care, and health care and systems research has been recognized at many levels, including by funding agencies. However, there is little guidance or established best practices on how to include non-academic reviewers in the grant review process. Here we describe our approach to the inclusion of people with lived experience in every stage of the grant review process. After a budget was created for a specific call, a steering committee was created. This group included researchers, people with lived experience, and health systems administrators. This group developed and issued the call. After receiving proposals, stage one was scientific review by researchers. Grants were ranked by this score and a short list then reviewed by people with lived experience as stage two. Finally, for stage three, the Steering Committee convened and achieved consensus based on information drawn from stages one and two. Our approach to engage people with lived experience in the grant review process was positively reviewed by everyone involved, as it allowed for patient perspectives to be truly integrated. However, it does lengthen the review process. The proposed model offers further practical insight into including people with lived experience in the review process.

## Background

People with lived experience are individuals who have first-hand experience of the medical condition(s) under consideration. This may be someone who has been diagnosed with the condition themselves or a caregiver of someone diagnosed with the condition. The value of including the perspectives of people with lived experience in health policy, health care, and health care and systems research has been recognized at many levels, including by funders. However, there is little documented guidance or best practice on how to include non-academic reviewers in the peer review process [1]. There may be no universal guidance, as each project should consider the most appropriate type of experience, both people who have been treated for the condition and care-takers have important perspectives on research priorities. Previous research in breast cancer has found that the incorporation of people with lived experience in the grant funding review process was considered beneficial both by the academic reviewers and the people with lived experience [2–5]. In the example in this report – the participants have first-hand experience with mental illness or addictions.

The literature has documented various ways that inclusion of only academic scientists in the review process is problematic. First, evidence shows that using only scientists in peer review often results in a bias against novelty [5]. Bias has a major influence on funding decisions, and the impact of this particular reviewer bias is magnified by low funding levels [6]. Second, it has been shown that when investigators had similar measures of productivity, their chance of success increased if the funding panel contained a member of the same institution [7]. Third, applications that are similar to the interests of the reviewers are often favored [8] and yet reviewed more critically [5], so they may be viewed as more important and also given more useful feedback due to the extent of expertise in the area. Put simply, reliance on scientists alone often results in ‘echo-chamber’ science, sacrificing novelty and innovation. Indeed, in the only study to compare reviewer scores directly, Fleurence et al. [9] found that prior to face to face meetings, reviewer scores varied significantly between scientists, patients, and stakeholders. This indicates that perspectives do indeed differ between such groups and that more consideration of other perspectives can enrich the process.

Including people with lived experience in research raises multiple ethical considerations [10], which we were not able to completely resolve with our process. The Canadian Institute for Health Research (CIHR) has recently released their draft guidance document for developing research partnerships with people with lived experience [10] with a focus on concern for the welfare of others, justice, and respect. This document focuses on the level trust needed between the team members, which may be hard to quantify but is easy to recognize when it is not present. A cornerstone of that trust is a mutual respect for different ways of knowing and interacting. Without that, it is difficult to capture the diversity of perspectives. There is an inherent power imbalance with including people with lived experience in the research process. This can create a barrier to participation if not managed well. Second, the shared commitment to achieving the common goal must be clear, with members feeling solidarity with each other and experiencing reciprocity. As engagement with people with lived experience can legitimize or add credibility to the research conducted, the process used must allow for full participation by the people with lived experience, avoiding tokenism. Other groups have identified specific actions that have ethical considerations, such as people with lived experience not receiving compensation for their time and work [11]. While none of the committee members received compensation for membership specifically, all others were participating as part of their employment. This is not equitable and also creates bias in which people with lived experience have the ability to participate, as many people are unable to volunteer their time. The impact of this bias is unknown but probably results in some perspectives not being included in the decisions.

Alberta Health Services, the provincial health care provider in Alberta, prioritizes patient centered care and innovation [12]. To that end, they created multiple Strategic Clinical Networks™ which are groups of clinicians, researchers, and patients who work together to bring innovation to front line practice and improve care. To further the influence of patient experience on research, the Addiction and Mental Health Strategic Clinical Network™ (AMH SCN™) began engaging people with lived experience in the grant funding review process. Here we present the approach used by the AMH SCN to incorporate the values and perspectives of people with lived experience in the grant review process for a provincial funding opportunity. We have also incorporated their comments and feedback into this article, to present the experience from all perspectives.

## Main text

### Background of the specific funding opportunity

The AMH SCN facilitated a research funding call connected to the Valuing Mental Health (VMH): Next Steps [13, 14], a document released by the Alberta Government in June 2017. This call specifically addresses identification of evidence-informed research practices and programs to improve community-based system integration in the addiction and mental health sector in Alberta with a focus on implementing programs rather than basic science investigations.

**Fig. 1:**
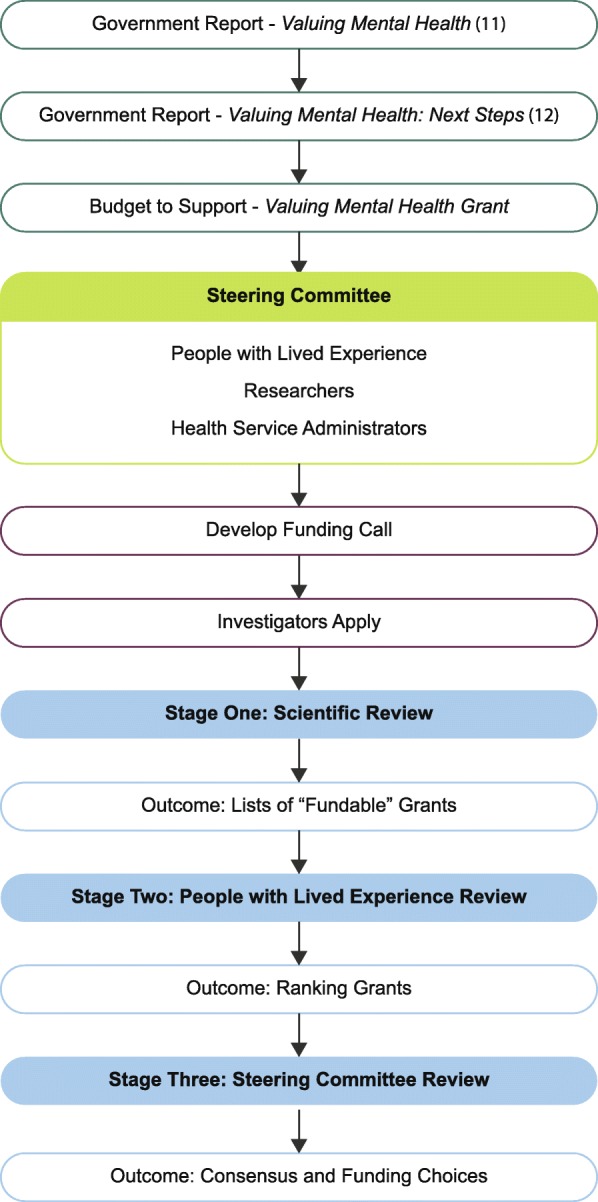
Schematic showing a summary of the grant development and review process

The call was titled the VMH Innovation and Integration Research Grant and a steering committee was created from experts in grant calls and in addiction and mental health research. The AMH SCN believes that people with lived experience are experts in addiction and mental health research, as they are best situated to understand the most impactful questions and gaps in knowledge. As such there were multiple people with lived experience invited to participate on the steering committee and involved as meaningful partners in the granting process at all stages - from designing through to awarding the grants. See Fig. 1 for summary of process. This included committee membership, decisions regarding the call design, and the weighing of domains for the scientific peer review. The steering committee also decided the final award discussion would consider ranking by a small group of individuals who have lived experience with addiction or mental health disorders and treatment. The committee thought this would increase the likelihood that the funded applications are of importance and relevance to the community. It also provided the people with lived experience an opportunity to provide feedback on the top applications in a less intimidating environment than relying on verbal feedback in the final meeting, as the ranking was compiled by the steering committee coordinator and presented anonymously at the meeting.

Alberta Health Services benefits from a Provincial Advisory Council on Mental Health and Addiction. This is a long-standing council, founded in 2012, who serves to advocate, advise, and reflect patient perspectives to improve the Addiction and Mental Health System. Council members have exposure to a broad array of issues, disease specific needs, and connection to different networks and communities. Volunteers from the Provincial Advisory Council came forward to participate in this grant process. While their prior experience on the committee may bias the group, it also ensured that the people with lived experience had support if they had any concerns with the process. This also demonstrated their ability to work within the health system and simplified the ethical considerations around consent, screening and recruitment of people with lived experience as they had been through extensive training with the Provincial Advisory Council. People with lived experience are often concerned that their judgments would not be taken seriously by scientists [4]. By engaging people with lived experience in both the design and review phase, this concern can be mitigated as the input in both helps to centre their perspectives as equal in the overall process. When people with lived experience are only involved in the review of applications, the opportunity to shape the call and therefore prioritize the research questions addressed, is missed. Furthermore, the breadth of disorders included in the addiction and mental health fields means that the specifics of the disorders in the grant applications could not be anticipated. This led to the people with lived experience who participated not having experience specific to the disorders in applications (necessarily, they were never asked to disclose their specific lived experience), rather they were people who volunteer in the Alberta health care system and have experience representing the entire community of people with lived experience in addiction and mental health treatment and operating in committee environments.

### Model for contribution to the review process

#### Stage one – scientific review

All applications had typical scientific peer review. Three independent experts (i.e., academic researchers) reviewed and scored each application on areas such as scientific merit, team strength, methodology and relevance. The AMH SCN contracted the administration of the call with Policy Wise for Children & Families. This is a not-for-profit organization in Alberta that works to develop and integrate evidence to inform, identify, and promote effective public policy and service delivery to improve the well-being of children, families, and communities. They received the reviews and calculated scores for the set of 22 applications. The steering committee had determined that only those scored above 75% of the total possible would be eligible for funding, this was to ensure that only applications with strong feasibility and methodology would be funded.

#### Stage two – people with lived experience review

The ten applications with the highest scores after scientific peer review that were also above the ‘fundable’ cut off point, were forwarded to three people with lived experience. They were asked to rank the applications from 1 to 10 with 1 being the highest priority from the perspective of people with lived experience. They were explicitly informed to exclude any applications that they did not believe will have significant positive impact but were asked to specify that they did so purposefully. The resulting three lists were compiled into one by a member of the AMH SCN. Significant discord was to be discussed amongst the people with lived experience during a separate meeting. If consensus could not be reached, the plan was to present the multiple perspectives to the steering committee rather than forcing agreement. However, this did not happen during this grant review process.

The people with lived experience were provided the entire application. However, they were not asked to review the scientific methodology or literature, nor were they required to review the entire application. They were asked to review the abstract and proposal to determine their rankings. This did not prevent some of the reviewers from being concerned about the impact of their self-reported lack of expertise in methodology. However, as with most grant reviews, that knowledge was over-represented at the review table.

When establishing their ranking, they were asked to consider the following criteria: (1) Does this project address a gap that people with lived experience view as important (it has impact on their lives)? (2) If the project is successful, will it address the gap and improve the community’s experience living with addictions and/or mental health disorders? (3) Is there any part of the project that is not feasible from the perspective of people with lived experience? (Example: recruitment will be very tough because most people with disorder ‘X’ will be excluded by ‘Y’). The people with lived experience were able to include notes with their lists in order to ensure their voices were ‘at the table’ even if they could not attend the final meeting as two of the three had conflicts and were unable to attend.

A practical aspect of this process is the need for additional time between scientific peer review deadlines and final steering committee decision making meetings. In this specific case, the people with lived experience agreed that they could review and prioritize in a week, which is a very short timeframe and when planning, more time should be allocated. Negotiating time with the people with lived experience is important, as this is usually done outside of both work and usual personal lives. Feedback from those involved was that this timeframe was reasonable and they appreciated the time they had.

In light of the grant review timeline, there was insufficient time for the three people with lived experience reviewers to meet and discuss discrepancies in their rankings. This was due to the fact that they all were employed in full time positions and were volunteering their time, videoconferencing was used for all meetings to lessen the burden of travel. In further grant calls time should be built into the process to address this need.

#### Stage three – steering committee review

The prioritized list from the people with lived experience was brought to the final steering committee meeting alongside the scientific peer-review scores and reviewer comments, for input into the final funding decisions. Discussion began with the highest scored application based on scientific peer-review, at the same time consideration of how high the people with lived experience prioritized that application was able to affect final funding decisions. Consensus was reached on the first to be funded, with that project’s budget subtracted from the total amount. Discussion at this level is critical in developing closer agreement [9]. This process was repeated until the ‘next to be funded’ exceeds available funding, then discussion moved to those that can be funded by available funding. Throughout the discussion the committee co-chairs made a point of involving people with lived experience and any discrepant ratings were also discussed. This was important in keeping with the spirit of true contribution and participation for people with lived experience.

### Suggestions/feedback from the involved people with lived experience

While the rubric for the people with lived experience was considered helpful, it was highlighted that having input into the design of the rubric would have enriched the process and ensured that the language was non-scientific. If time allows, it may also be useful to pair the person with lived experience with a mentor from the scientific review community and provide an example of a grant application that can be read together. This could provide time for the people with lived experience to familiarize themselves with typical grant sections and language outside of meeting times, which are more intimidating situations. It was interesting that all of the researchers who participated in the steering committee communicated the value the people with lived experience brought to the discussions to the chair of the committee, this could be expanded upon in the future through the proposed mentorship. Feedback also demonstrated that people with lived experience were concerned that their reviews would be biased and that may have led to unnecessary pressure on themselves. Several of the people with lived experience who reviewed grants asked that in the future, they be provided with feedback on their reviews after the process is finished. Completing the circle and providing feedback to the people with lived experience on their grant reviews was also suggested.

## Conclusions

This is one process to engage people with lived experience in grant review that was positively reviewed by everyone involved in the process. On the positive side, it allows for the patient perspectives to be truly integrated. On the negative side, it does add time to the review process. The limitations of this model include the following: (1) This was a small provincial funding opportunity. The question remains if this process would scope and scale to national/international levels (i.e., > 1000 applications). (2) We did not offer compensation for participation in the review process. This almost certainly biased who was able to participate (i.e., they could afford the time involved). How best to compensate people with experience for participation in research remains an unresolved question. (3) We did not have a formal evaluation process as part of this initiative. Future efforts should include a qualitative analysis that engages people with lived experience, the academic and health administrators involved, and those applying for the funding. The proposed model does offer further insight into including people with lived experience in the review process, building on a nascent literature base [2–4, 9, 15].

## Data Availability

Not applicable.

## Abbreviations

AMH SCN™: Addiction and Mental Health Strategic Clinical Network™
VMH: Valuing Mental Health
